# Denitrifiers Make Great Contribution to Antibiotic Resistance Genes Dissemination in the Gut of Earthworms

**DOI:** 10.3390/ijms27020797

**Published:** 2026-01-13

**Authors:** Maria Rafraf Ali, Yongjing Chen, Mingjun Li, Muhammad Jafir, Mamona Rafraf Ali, Guowei Zhou, Qingye Sun

**Affiliations:** 1 Anhui Province Engineering Laboratory for Mine Ecological Remediation, School of Resources and Environmental Engineering, Anhui University, Hefei 230039, China; maria.chemist1@gmail.com (M.R.A.); chenyongjing@shcac.edu.cn (Y.C.); m.jafir.uaf@gmail.com (M.J.); 2 Anhui Province Key Laboratory of Wetland Ecological Protection and Restoration, School of Resources and Environmental Engineering, Anhui University, Hefei 230039, China; 3 Shandong Key Laboratory of Water Pollution Control and Resource Reuse, School of Environmental Science & Engineering, Shandong University, Qingdao 266237, China; 202320541@mail.sdu.edu.cn; 4 School of Energy Science and Engineering, University of Science and Technology of China, Hefei 230026, China; rafraf158@gmail.com; 5 Guangzhou Institute of Energy Conversion, Chinese Academy of Sciences, CAS Key Laboratory of Renewable Energy, Guangdong Provincial Key Laboratory of New and Renewable Energy Research and Development, Guangzhou 510640, China

**Keywords:** gut microbiota, earthworm, denitrification, ARG spread

## Abstract

Antibiotic resistance genes (ARGs) pose a serious threat to the environment worldwide. The guts of soil animals are a hotspot for ARGs and denitrification in soils. However, it is unclear how denitrification affects the spread of ARG in the earthworm’s gut. In this study, the typical soil earthworm *Pheretima guillelmi* was employed, and was used for performing anoxic incubation with gut content amended with nitrate and nitrite. To analyze the data, a combination of chemical analysis, 16S rRNA-based Illumina sequencing, and high-throughput qPCR were employed. Nitrate treatments, particularly at 5 mM, caused substantial reductions in nitrate concentrations, with a corresponding increase in nitrite, nitrous oxide (N_2_O), and nitric oxide (NO) emissions compared to the treatments with the addition of 1 and 2 mM nitrate. Nitrite (0.2, 0.5 and 1 mM) amendments also enhanced the accumulation of nitrogen intermediates. Organic acid production, including acetate and pyruvate, was the highest under the 5 mM nitrate treatment. This treatment also promoted the highest level of glucose utilization, suggesting that glucose metabolism supports enhanced organic acid production. Both nitrate and nitrite treatments exhibited the pronounced enrichment in ARGs, particularly for beta-lactam and multidrug resistance genes. Denitrifying bacteria such as *Aeromonas*, *Bacillus*, *Raoultella*, and *Enterobacter* were identified as key hosts for these ARGs. These results emphasized that denitrifying bacteria play a pivotal role in the horizontal transfer of ARGs, underscoring the need for careful nitrogen management in agricultural practices to control the spread of antibiotic resistance in natural environments.

## 1. Introduction

Antibiotic resistance genes (ARGs) are an emerging threat to human and environmental health. The entire ecosystem is being disturbed by the excessive use of antibiotics and fertilizers [1,2,3]. The use of these fertilizers results in noticeable alterations in soil chemistry and microbial composition [4]. The accumulation of excessive nitrogen, mainly as nitrates and nitrites, disrupts the normal nitrogen cycle and causes changes in the distribution of microbial populations [5]. Such conditions promote the activity and proliferation of denitrifiers, which play a critical role in the nitrogen cycle. However, these microbes not only contribute to nitrogen removal but also serve as reservoirs for antibiotic resistance genes. These genes spread into the environment, contaminating soil, water, and even the digestive systems of animals [6,7]. ARGs can move between environments and along food webs, posing a significant risk to public health, agriculture, and the environment [8,9,10,11]. Antibiotic resistance genes (ARGs) have significant potential for widespread transmission [12]. The primary mechanism responsible for the swift spread of ARGs is horizontal gene transfer, which is facilitated by mobile genetic elements (MGEs). This process accelerates the emergence and global spread of antibiotic resistance [13,14], positioning antibiotic resistance as one of the most pressing threats to human health in the 21st century. Researchers have documented that long-term fertilization promotes the dissemination of ARGs in soils and in certain soil fauna, such as earthworms [15].

Earthworms play a crucial role in maintaining soil health. The gut microbes of the earthworm play a vital role in promoting soil health by enhancing soil fertility and structure [16,17]. In addition to these benefits, the intestinal microflora is involved in the degradation of organic matter, which further contributes to nutrient cycling and soil enrichment. The gut contains a wide variety of denitrifiers within an anoxic environment [18]. Nitrate addition can stimulate specific microbial processes, such as denitrification, which may alter the composition of the microbial community within the earthworm gut. Previous studies highlighted that the gut nitrate reduction processes, coupled with nitrate reducing bacteria, shape the gut microbial community structure and exert a selective pressure on gut microbes [19]. Microbial populations assimilate ARGs because some organisms are able to survive in nitrate rich environments and possess the trait of incorporating or enhancing resistance. Hence, varied microbial activity in the presence of nitrate in the gut may alter the dynamics of ARGs.

The earthworm gut, serving as a unique microbial habitat, represents a potential reservoir for ARGs [20]. It is worth noting that earthworms can also ingest ARGs from their diet, and these ARGs can subsequently be incorporated into the gut microbes of these organisms [18,19,20]. However, natural microbes in the gut can block the flow of some of these genes [21]. It has been previously established that the gut of the earthworm is enriched with a greater number of ARGs than the surrounding surface soil [22]. However, earthworms can either suppress or amplify resistance depending on the circumstances, for example, ref. [23] demonstrated that the microbes within the gut of the earthworm could minimize the risk of ARGs spread. Recent studies also have considered earthworms a possible carrier to transfer ARGs between the soil and the gut bacteria [17,21,24], yet, the role of gut microbiota in this process remains elusive [25]. Therefore, further studies are required to explore how the gut environment of earthworms, particularly in relation to denitrifying bacteria, may contribute to the accumulation and spread of ARGs.

The ability of denitrifying bacteria in the nitrogen cycle is well established. However, more recent studies show these bacteria may also contribute to the persistence and propagation of ARGs in the environment. Some studies indicate that these microorganisms may enable horizontal gene transfer, thereby increasing the dissemination of ARGs and integrating the spread of resistance with the removal of contaminants [26]. This reflects the importance of denitrifiers as potential carriers of ARGs in both engineered and natural systems. To date, a small number of studies have investigated the denitrifier-mediated transfer of ARGs in soils and aquatic systems, but their role in microbiomes associated with animals remains mostly unexplored.

As a mobile anoxic mini-environment, the earthworm gut has a big potential for denitrification and provides an ideal habitat for denitrifiers [27]. These denitrifiers, which are mainly members of the phyla *Proteobacteria* and *Firmicutes*, are essential to nitrogen cycling in these ecosystems, but their interaction with mobile genetic elements (MGEs) raises serious concerns about the spread of ARGs [28]. It is becoming more and more evident that denitrifiers may act as vectors for the spread of ARGs [29,30,31]. Several denitrifying bacterial species are known to be able to acquire and spread ARGs through horizontal gene transfer (HGT) mechanisms, especially in gut microbiomes [30,31,32]. Mobile genetic elements (MGEs), such as plasmids and transposons, which facilitate the transfer of resistance traits between bacterial genera, play a crucial role in the dissemination of ARGs [31,32,33]. Considering denitrifiers potential hosts of ARGs within the gut of earthworms is a unique ecological approach. This perspective may aid understanding in the field of nitrogen-transforming bacteria as concealed pathways of antibiotic resistance and underscores the originality and ecological importance of denitrifiers in gut-associated ecosystems [27]. The ability of denitrifiers to transport ARGs poses a risk to ecosystems and public health as they colonize a variety of gut environments. However, the contribution of denitrifiers to the hosts of ARGs and then accelerating ARG enrichment is underexplored.

In this study, we examined dissemination of ARGs within the earthworm gut after adding nitrate and nitrite as substrates during the anoxic incubation. Combining chemical analysis, 16S rRNA-based Illumina sequencing and high-throughput qPCR, we aim to achieve the following: (1) investigate the impact of denitrification on ARG enrichment in the earthworm gut; (2) identify the abundances and bacterial composition of denitrifiers involving in ARG dissemination. Hence, this study will increase the understanding of antibiotic resistance development in connection to environmental nitrogen cycling in soils.

## 2. Results

### 2.1. Time Course of Nitrate, Nitrite, Nitrous Oxide, and Nitric Oxide During the Incubation

**Figure 1:**
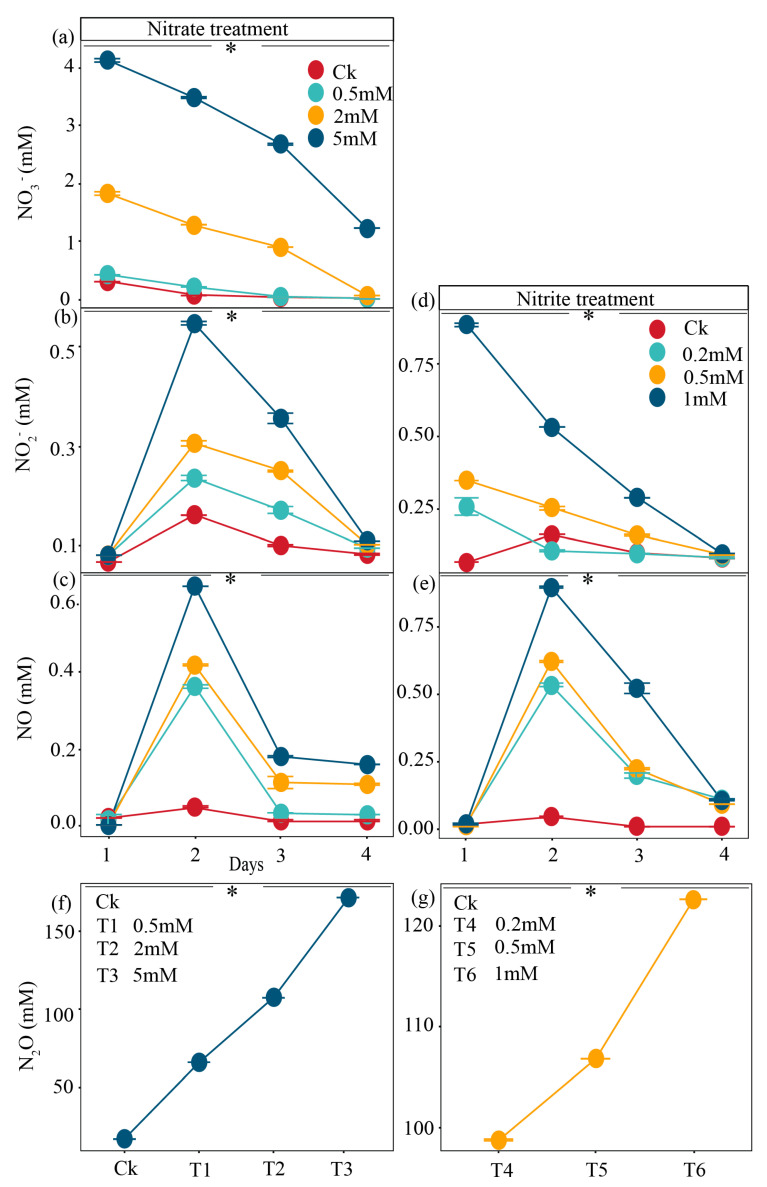
Dynamics of the concentrations of (**a**) NO_3_^−^, (**b**,**d**) NO_2_^−^, (**c**,**e**) NO, and (**f**,**g**) N_2_O during microcosmic incubation with nitrate and nitrite treatments. The concentrations of NO_3_^−^, NO_2_^−^, and NO were measured over days 1, 2, 3, and 4, while the concentration of N_2_O was measured at day 4. Data points represent the mean ± standard deviation (*n* = 3), as indicated by error bars, and statistical significance was assessed using ANOVA (*p* < 0.05). The asterisk (*) in the figure indicates that the overall treatment effect was statistically significant. Treatments T1, T2, and T3 correspond to nitrate concentrations of 0.5 mM, 2 mM, and 5 mM, respectively, while T4, T5, and T6 correspond to nitrite concentrations of 0.2 mM, 0.5 mM, and 1 mM, respectively.

For the nitrate treatments, the decrease in NO_3_^−^ concentrations exhibited dose- and time-dependent trends across all treatments with nitrate (Figure 1a). The 5 mM nitrate treatment exhibited the most substantial depletion, declining from an initial concentration of approximately 4.0 mM to nearly 1.2 mM by the end of the incubation period (Figure 1a). In the 0.5 mM and 2 mM treatments, nitrate was almost completely consumed within the first several days (Figure 1a). Concurrently, nitrite (NO_2_^−^) accumulation was observed as a transient intermediate during nitrate reduction (Figure 1b). The 2 mM and 5 mM nitrate treatments led to a notable increase in nitrite, peaking at around 0.5 mM and 0.3 mM on day 2, respectively, before declining toward the end of the incubation (Figure 1b). In contrast, the 0.5 mM treatment and the control showed minimal nitrite accumulation (Figure 1b). Nitrous oxide (N_2_O) and nitric oxide (NO) emissions were also influenced by nitrate amendment (Figure 1c,f). After transient accumulation on day 2, NO was rapidly consumed during the incubation (Figure 1c). N_2_O production was significantly enhanced in the 5 mM and 2 mM nitrate treatments, reaching maximum values of around 170 mM and 110 mM, respectively (Figure 1f).

In terms of the nitrite treatments, the decreases in NO_2_^−^ concentrations were also dose- and time-dependent across all treatments with (Figure 1d). Furthermore, the trends in N_2_O and NO concentrations in the nitrite treatments were with those in the nitrate treatments (Figure 1). Statistical analysis using one-way ANOVA revealed significant differences among the treatment groups (*p* < 0.05; Figure 1). The asterisk (*) in the figure indicates that the overall treatment effect was statistically significant, as confirmed by the ANOVA results.

### 2.2. Turnover of Organic Acids During Incubation with the Earthworm Gut

**Figure 2:**
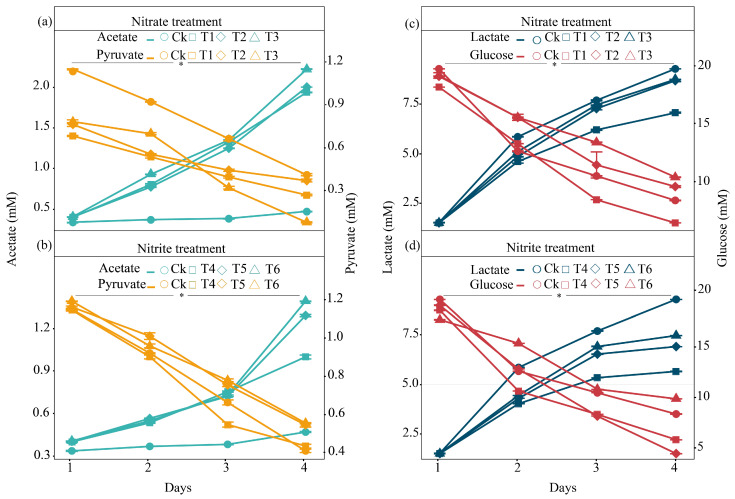
Temporal changes in the organic acid concentrations under nitrate and nitrite treatments during microcosmic incubation. (**a**,**b**) Acetate and pyruvate concentrations in (**a**) nitrate and (**b**) nitrite treatments over a 4-day period. (**c**,**d**) Lactate and glucose concentrations in (**c**) nitrate and (**d**) nitrite treatments. Data points represent the mean values ± standard deviation (*n* = 3), as indicated by error bars. Statistical analysis using one-way ANOVA revealed significant differences among the treatment groups (*p* < 0.05; Figure 2). The asterisk (*) in the figure indicates that the treatment effect was statistically significant.

The production of organic acids exhibited distinct trends over the four-day period. Acetate production increased progressively, reaching its maximum on day 4 under the 5 mM nitrate treatment. In contrast, pyruvate production declined between day 1 and day 4, with the highest levels recorded on day 4 under the 1 mM nitrite treatment (Figure 2a,b). Glucose production followed a similar trend, peaking on day 1 and decreasing on subsequent days in the 1 mM nitrite treatment. The 5 mM nitrate treatment yielded the highest glucose concentration on day 3, followed by a slight decline on day 4 (Figure 2c). Overall, while most organic acids showed an increasing trend over time, and this increase was not uniform across all compounds, nitrate treatments, particularly at higher concentrations, consistently yielded the highest levels of organic acids (Figure 2). Statistical analysis using one-way ANOVA revealed significant differences among the treatment groups (*p* < 0.05; Figure 2). The asterisk (*) in the figure indicates that the treatment effect was statistically significant.

### 2.3. ARG Dissemination in the Earthworm’s Gut: Variations Among the Control, and the Nitrate and Nitrite Amendments

**Figure 3:**
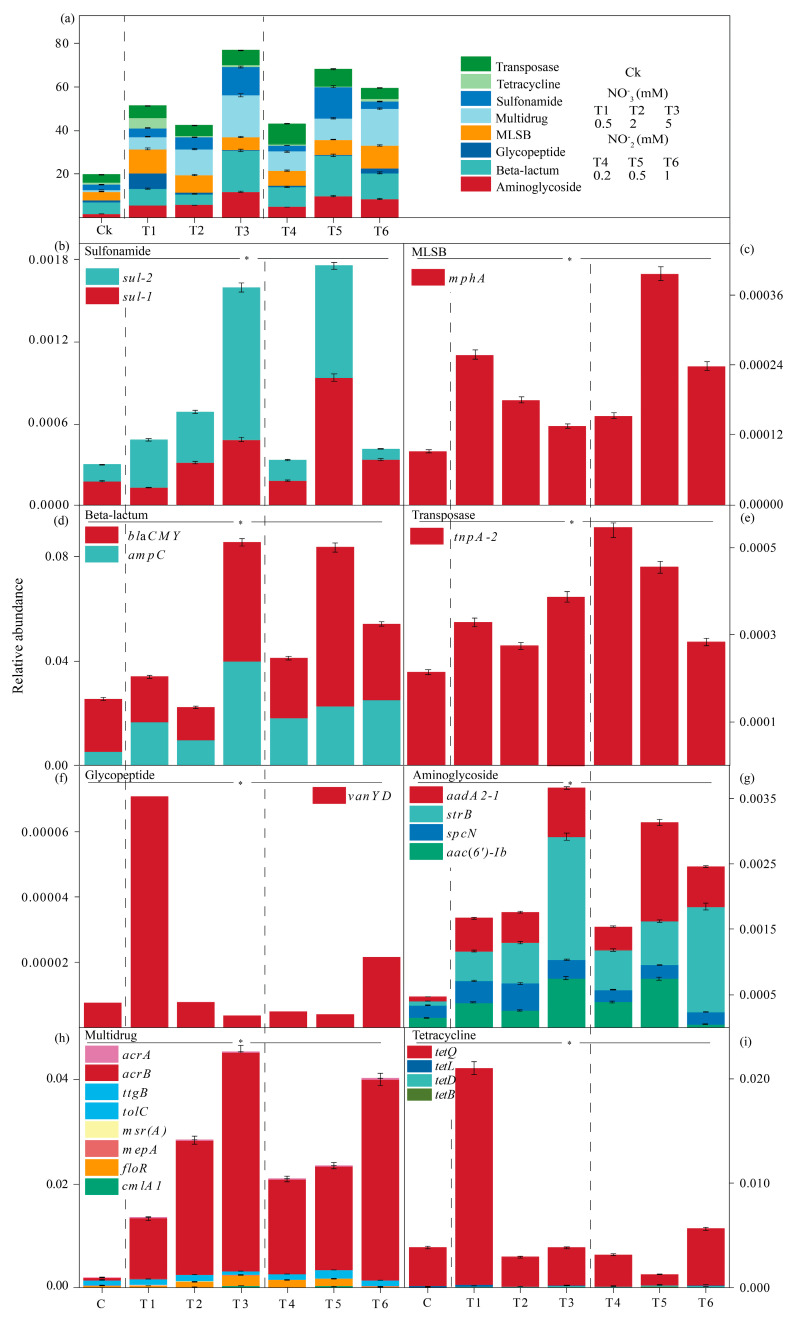
Relative abundance of antibiotic resistance genes (ARGs) and transposase across different categories under nitrate and nitrite treatments. (**a**) Overall abundance of ARG categories, (**b**) sulfonamide, (**c**) MLSB, (**d**) beta-lactam, (**e**) transposase, (**f**) glycopeptide, (**g**) aminoglycoside, (**h**) multidrug, and (**i**) tetracycline. Mean ± standard deviation, presented with error bars. Statistical analysis using one-way ANOVA revealed significant differences among the treatment groups (*p* < 0.05; Figure 3). The asterisk (*) in the figure indicates that the overall treatment effect was statistically significant, as confirmed by the ANOVA results.

A total of 23 antibiotic resistance genes (ARGs) from eight different categories were studied in earthworm gut microcosms. Statistical analysis using one-way ANOVA revealed significant differences among the treatment groups (*p* < 0.05; Figure 3). The asterisk (*) in the figure indicates that the overall treatment effect was statistically significant. Sulfonamide resistance (Figure 3b) was significantly affected by both nitrate and nitrite treatments. *Sul-1* and *sul-2* showed a marked increase, especially under nitrite treatments. In 1 mM nitrite treatment, *sul-1* expression increased notably compared to that in the control (Figure 3b). In terms of the beta-lactam resistance, *ampC* and *blaCMY* displayed considerable upregulation in both nitrate and nitrite treatments (Figure 3d). The gene *ampC* showed the highest expression in the 1 mM nitrite treatment, while *blaCMY* reached its peak expression level in the 0.5 nitrite treatment (Figure 3d). Transposases such as *tnpA-2* were highly upregulated in both the nitrate and nitrite treatments, with the strongest increase observed under 0.2 mM nitrite (Figure 3e). In aminoglycoside resistance, *aac(6′)-Ib*, *spcN*, and *strB* exhibited significant upregulation across all treatments (Figure 3g). The most pronounced increase was observed for *aac(6′)-Ib*, which peaked in the 1 mM nitrite treatment (Figure 3g). Similarly, *strB* reached its highest expression under 5 mM nitrate, reflecting a dose-dependent response (Figure 3g). Nitrite amendments, especially at higher concentrations, were particularly effective in promoting aminoglycoside resistance. The multidrug resistance category showed a clear dose-dependent increase in *acrA*, *acrB*, and *floR* expression, with the highest relative abundance observed under 5 mM nitrate and 1 mM nitrite (Figure 3h). Tetracycline resistance genes, including *tetB*, *tetD*, *tetL*, and *tetQ*, also demonstrated an increase, especially in 0.5 mM and 1 mM (Figure 3i). The relative abundance of *tetB* was particularly enhanced, with higher levels observed under nitrite amendments than that under nitrate amendments (Figure 3i). In addition, categories with less significant roles in ARG expression included glycopeptide resistance (Figure 3f) and MLSB resistance (Figure 3c), while *vanYD* and *mphA* showed slight increases, particularly in 1 mM nitrite treatments, and their upregulation was less pronounced compared to other categories (Figure 3c,f). Similarly, *cmlA1* displayed stable expression across all treatments, with minor increases in both nitrate and nitrite treatments (Figure 3h).

### 2.4. Molecular Profile of Nitrogen Reduction Pathways in the Earthworm’s Gut

**Figure 4:**
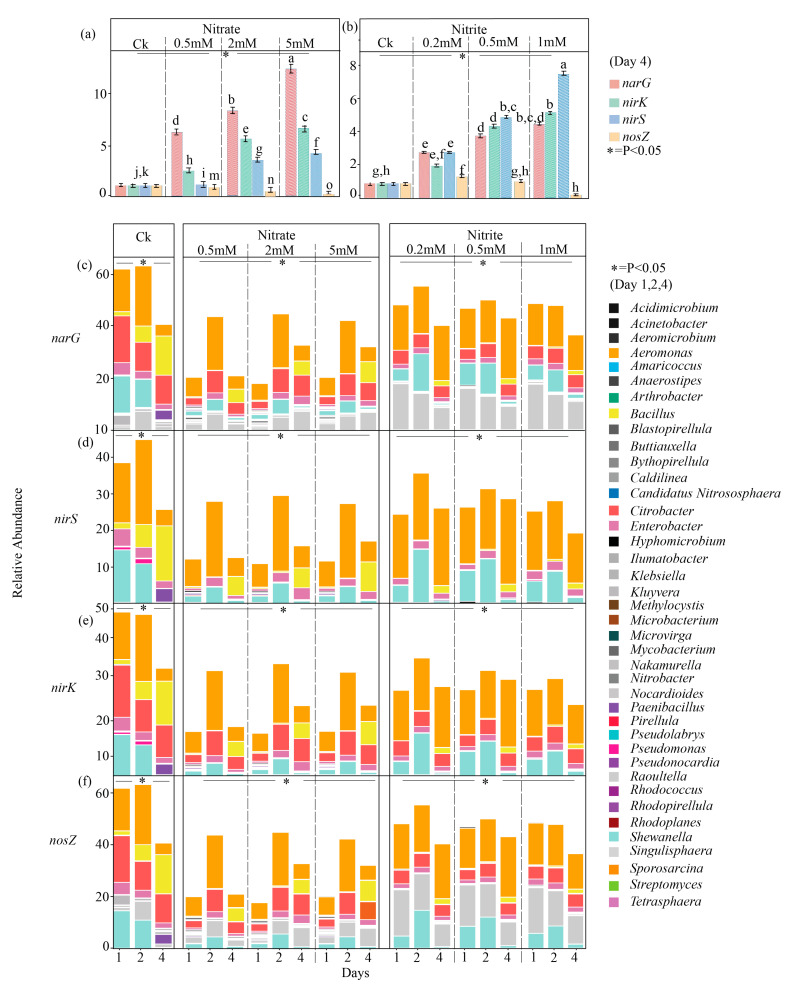
Relative abundance of denitrifying gene targets across nitrate and nitrite treatments during microcosmic incubation. (**a**,**b**) Gene abundance of *narG, nirK, nirS*, and *nosZ* in (**a**) nitrate and (**b**) nitrite treatments at day 4. Letters indicate significant differences among treatments. The figure shows the mean ± standard deviation, represented by the error bars. (**c**–**f**) Temporal changes in the abundance of denitrifier taxa, as quantified by 16S rRNA sequencing for (**c**) *narG*, (**d**) *nirS*, (**e**) *nirK*, and (**f**) *nosZ* over days 1, 2, and 4. The stacked bar plots show the relative abundance of different denitrifier genera, with the genera color-coded in the legend. Treatments are indicated by the following: Ck (control), T1-T3 (nitrate concentrations of 0.5 mM, 2 mM, and 5 mM, respectively), and T4-T6 (nitrite concentrations of 0.2 mM, 0.5 mM, and 1 mM, respectively). Statistical analysis using ANOVA revealed significant differences among the treatment groups (*p* < 0.05; Figure 4). The asterisk (*) in the figure indicates that the overall treatment effect was statistically significant.

The gene *narG*, associated with nitrate reduction, showed a gradual increase across both nitrate and nitrite treatments, with the highest abundance observed under 5 mM nitrate (Figure 4a). However, the abundances of *nirK* showed significant variation across treatments (Figure 4). Moreover, *nirS* followed a similar pattern, with the highest expression in the treatment with 1 mM nitrite (Figure 4b). The abundance of the gene *nosZ* demonstrated a contrasting tendency to that of *nirK* and *nirS* (Figure 4) in the nitrate and nitrite treatments (Figure 4). There were 40 genera identified as the potential denitrifying bacteria in the nitrate and nitrite treatments (Figure 4c–f). Statistical analysis using ANOVA revealed significant differences among the treatment groups (*p* < 0.05; Figure 4a–f).

Among the denitrifying bacteria, *Aeromonas*, *Bacillus*, *Rautella*, *Shewanella*, *Paenibacillus*, and *Enterobacter* were the dominant denitrifying genera, potentially harboring *narG*, *nirK*, *nirS* and *nosZ* (Figure 4c–f). In summary, nitrate treatments promote the abundances of *Aeromonas* and *Shewanella*, while nitrite treatments favor increases in the abundances of *Raoultella* and *Shewanella*, with *Bacillus* and *Paenibacillus*, showing minimal variation (Figure 4c–f). In detail, for *Aeromonas*, its abundance was highest on day 2 and day 4 under the nitrate and nitrite treatments during the incubation, respectively (Figure 4c–f). The genus *Enterobacter* showed a notable increase in abundance during the incubation compared to the during the incubation, displaying a positive response to both nitrate and nitrite treatments (Figure 4c–f). Similarly, *Bacillus* abundance was promoted in both nitrate and nitrite treatments, being the highest on day 4 during the incubation (Figure 4c–f). *Raoultella* showed a significant increase in nitrite treatments, especially at 0.2 mM and 0.5 mM, whereas *Paenibacillus* remained at consistently low concentrations across both treatments (Figure 4c–f). The abundances of *Shewanella* peaked on day 2 in the 2 mM and 5 mM nitrate treatments and remained abundant in the nitrite treatments, particularly at 1 mM, with only a slight decrease on day 4 (Figure 4c–f). The LEFSe analysis confirmed the notable increase in these denitrifying genera under selective pressure, emphasizing their dual role. The LEFSe analysis also confirmed the notable increase in these denitrifying genera under selective pressure, emphasizing their vital function (Figure S3).

### 2.5. Understanding the Microbial-ARG Network: Composition and Interactions

**Figure 5:**
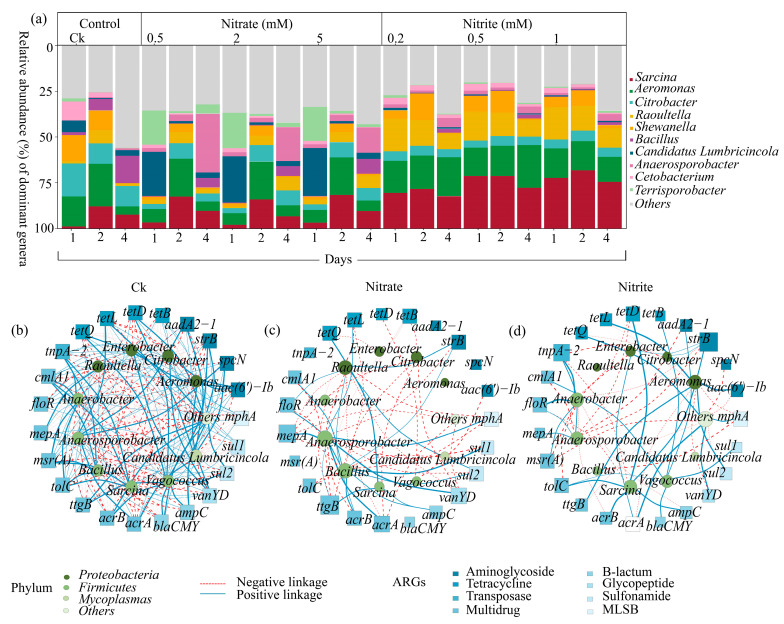
Genus variation and network analysis of antibiotic resistance genes (ARGs) across nitrate and nitrite treatments during microcosmic incubation. (**a**) Relative abundance of dominant genera on days 1, 2, and 4 across treatments: control (Ck), nitrate (0.5 mM, 2 mM, 5 mM), and nitrite (0.2 mM, 0.5 mM, 1 mM). The stacked bar plot shows the relative abundance (%) of dominant genera, with the genera color-coded, as indicated in the legend. (**b**–**d**) Spearman network analysis showing the correlation between genera and ARGs under (**b**) control, (**c**) nitrate, and (**d**) nitrite treatments. Nodes represent genera (green) and ARGs (blue), with positive linkages shown by solid lines and negative linkages indicated by dashed lines. These significant associations were determined based on spearman correlation analysis (*p* < 0.05), demonstrating significant correlations between genera and antibiotic resistance genes (ARGs), including aminoglycoside, tetracycline, transposase, multidrug, β-lactam, glycopeptide, sulfonamide, and MLSB.

The microbial communities among treatments were clearly separated according to the NMDS analysis (Figure S1b,c). In nitrate treatments, *Firmicutes* exhibited a similar pattern to *Proteobacteria*, the abundance of which increased on day 2 but decreased on day 4. In contrast to the control and nitrate treatments, nitrite treatments resulted in a rise in *Firmicutes* and a fall in *Proteobacteria* abundance (Figure 5a and Figure S1a). All treatments had the lowest index on day 2, while nitrate had the highest value among most of the index on day 4 (Figure S2).

In the control condition, *sul1* and *sul2* showed weak positive correlations with *Citrobacter* and *Enterobacter*, though they were non-significant (Figure 5b). The beta-lactam resistance gene *ampC* had negative correlations with *Bacillus* and *Raoultella* (Figure 5). The aminoglycoside resistance gene *aac(6′)-Ib* showed strong positive correlations with *Aeromonas*, *Citrobacter*, and *Enterobacter* (Figure 5b). Other genes like *acrA* and *mepA* showed non-significant trends in multi-drug resistance (Figure 5b). Under nitrate treatment, *sul1* and *sul2* had strong positive correlations with *Bacillus*, while *Raoultella* showed a minor role in sulfonamide resistance (Figure 5c). The gene *ampC* correlated significantly with *Bacillus* (Figure 5c). Aminoglycoside resistance, particularly that conferred by the *strB* gene, showed significant correlations with *Bacillus* (correlation = 0.683) and *Raoultella* (correlation = 0.750) (Figure 5c). Multidrug resistance genes such as *acrA*, *acrB*, and *floR* showed significant positive correlations with *Bacillus*, and *Raoultella* (Figure 5c). In the nitrite treatment, *sul1* and *blaCMY* showed strong positive correlations with *Aeromonas*, while *acrA* and *strB* correlated with both *Bacillus* and *Raoultella*, highlighting their roles in beta-lactam resistance (Figure 5d). Multi-drug resistance genes exhibited strong positive correlations with both genera, particularly *acrA* and *acrB*, and *ttgB* (Figure 5d). Spearman correlation analysis confirmed that these correlations were significant. *TnpA-2*-displayed notable associations with multiple resistance genes among the various genera, highlighting its role in gene transfer (Figure S4).

### 2.6. Denitrifying Taxa as an ARG Host: Correlation with Environmental Factors and Microbial Genera

**Figure 6:**
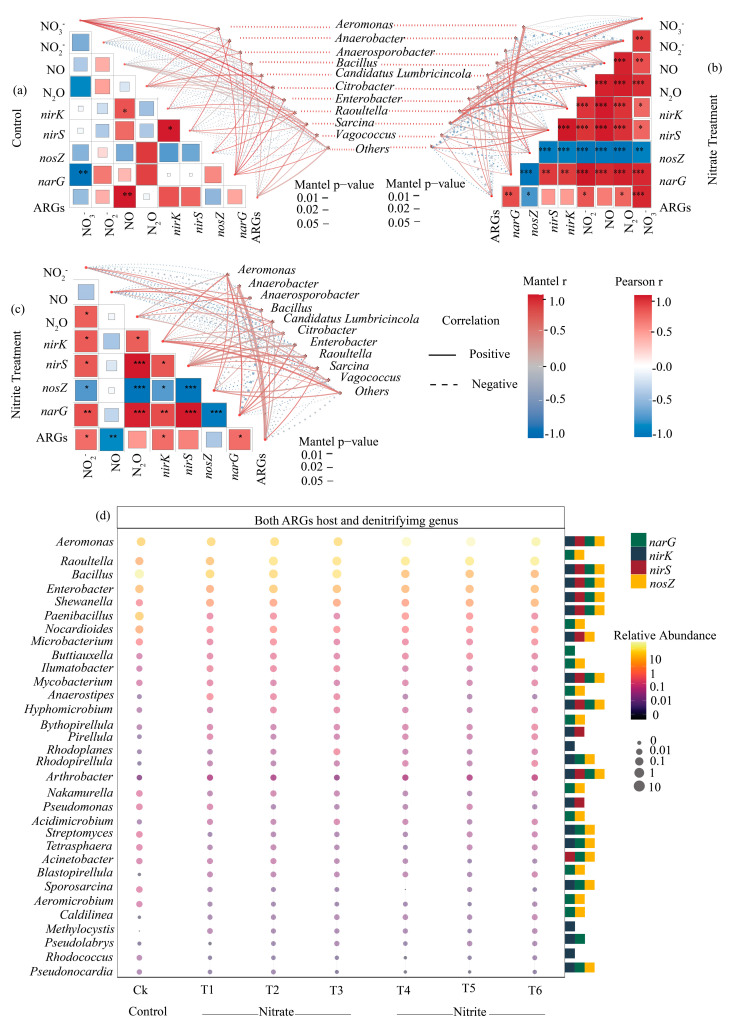
Mantel correlation analysis and co-occurrence of denitrifying genera and antibiotic resistance genes (ARGs) under nitrate and nitrite treatments. (**b**,**c**) Mantel correlation analysis showing the relationships between environmental factors and denitrifying genera across (**a**) the control, and the (**b**) nitrate and (**c**) nitrite treatments. Positive correlations are shown in red, while negative correlations are shown in blue. Statistical significance is indicated by asterisks (*p* < 0.05) * = ≤0.05, ** = ≤0.01, *** ≤ 0.001. (**d**) Bubble plot depicting the co-host of denitrifying genera and ARGs. The plot displays the relative abundance of genera (represented by bubble size and color intensity) that harbor both denitrifying genes and ARGs across treatments (Ck, T1–T3 for nitrate, and T4–T6 for nitrite). The color scale represents relative abundance, ranging from 0 to 10, with darker shades indicating higher abundance.

There were 10 genera significantly correlated with denitrification-related intermediate concentrations and gene abundances (Figure 6c). Statistical significance is indicated by asterisks, where *p* < 0.05: * = ≤0.05, ** = ≤0.01, and *** = ≤0.001. For the abundance of *Aeromonas*, positive associations were observed with the concentrations of NO_2_^−^ and NO, as was the case for the abundances of *nirK*, *nirS*, and *narG* under both the nitrate and nitrite treatments (Figure 6b,c). In addition, it resulted in positive associations between the abundance of *Bacillus* and the concentrations of NO_2_^−^, NO, and N_2_O, as well as between the former and *nosZ* abundance (Figure 6b). Under nitrite treatment, however, the associations shifted, with significant correlations found with NO_3_^−^, N_2_O, *nirS*, *nosZ*, and *narG* (Figure 6c). In comparison, *Enterobacter* showed positive associations with NO, *nosZ*, and *narG* in the nitrate treatments (Figure 6b). However, it led to stronger associations with NO_2_^−^, N_2_O, *nirK*, *nirS*, *nosZ*, and *narG* in nitrite treatments (Figure 6c).

## 3. Discussion

### 3.1. Earthworm Gut Has Big Potential for Denitrification

Both nitrate and nitrite are crucial substrates in the denitrification process. In this study, reductions of nitrate into nitrite and then of nitrite into NO and N_2_O were detected in both the nitrate and nitrite treatments (Figure 1), indicating the occurrence of denitrification in the gut of the earthworm. Consistently, the abundances of denitrification-related genes such as *narG*, *nirK*, *nirS*, and *nosZ* showed dose- and time-dependent trends in both nitrate and nitrite treatments, sharing the same characteristics with nitrate and nitrite during incubation [34] (Figure 4) This implies that microbial communities were actively reducing nitrate into nitrite and subsequently into N_2_O. In detail, abundant microorganisms were identified to be involved in denitrification during incubation (Figure. 4). Among them, the genera, including *Aeromonas* [35], *Bacillus* [36], *Shewanella* [37], *Paenibacillus*, and *Enterobacter* [38], carry all of the denitrification-linked genes containing *narG*, *nirK*, *nirS*, and *nosZ* (Figure 4). Most of them displayed the highest abundances on day 2 in the treatments with nitrate during incubation (Figure 4), corresponding to the time course of nitrate concentrations. This further indicates the reduction of nitrate into nitrite, NO, N_2_O, and even N_2_ during incubation. Likewise, these genera were also abundant in the nitrite amendments (Figure 4), showing a similar composition and tendency to the genera in the nitrate setups. Although the total abundances of denitrifiers in the nitrate/nitrite treatments were lower than those in the control, they still accounted for as high as 35~50% (Figure 4). Moreover, the nitrate and nitrite treatments showed a strong correlation between *Aeromonas*, *Bacillus*, and *Enterobacter*, as well as the genes associated with denitrification (*nirK*, *nirS*, *nosZ*, and *narG*) (Figure 6). These findings suggest that the earthworm gut was the hotspot for denitrification and harbored abundant denitrifying bacteria, consistent with the results of the previous study [39,40,41].

### 3.2. Denitrification Process Stimulates ARG Dissemination

With increasing concentrations of nitrate and nitrate, the total abundances of the detected ARGs were elevated during the incubation, especially in the nitrate treatments (Figure 3). Furthermore, significant relationships were found between ARG abundances and concentrations of NO_3_^−^/NO_2_^−^/NO/N_2_O in the nitrate treatments (Figure 6). These indicated the stimulative impact of denitrification on ARG enrichments. Furthermore, the abundances of sulfonamide, beta-lactam, aminoglycoside, transposase, multidrug-involving genes, were markedly enriched during the incubation (Figure 3). The variations in bacterial growth of these categories of ARG hosts (such as *Bacillus* and *Aeromonas*) for nitrate treatment were concentration-dependent (Figure 4), with each species responding differently. As indicated in previous research, *Bacillus* species are known for their denitrification capabilities, which are optimized under higher nitrate concentrations. This metabolic activity of denitrification can lead to increased oxidative stress. It has been well documented that high nitrate concentrations can lead to increased oxidative stress and the production of reactive oxygen species (ROS) during denitrification processes [42]. This stress can activate bacterial defense mechanisms, including a relative abundance of ARGs and the prompting of bacteria to acquire ARGs as well [42,43]. In addition, high nitrate concentrations can favor the proliferation of *Bacillus* strains, which could contribute to an increased prevalence of ARGs in the microbial community [44]. However, the abundances of MLSB, glycopeptide and tetracycline-linked ARGs exhibited different patterns, with *Pirellula* hosting MLSB, *Methylocystis* hosting MLSB and glycopeptide, and *Pseudomonas* hosting tetracycline-linked ARGs in these categories (Figure 3). The genera *Pirellula*, *Methylocystis*, and *Pseudomonas* showed a concentration-dependent decline with increasing nitrate levels due to nitrite stress [45]. Elevated nitrate concentrations can cause toxic nitrite accumulation, leading to physiological stress and competition among these nitrate-reducing microbes, thus potentially contributing to a reduction in the abundance of these ARGs *Caldilinea*, which exhibits complex responses to nitrate and may also be affected by shifts in microbial community dynamics that influence the distribution of these resistance genes [46]. Thus, changes in microbial composition due to varying nitrate levels can disrupt denitrification and alter both nitrogen cycling and ARG profiles [47]. Meanwhile, the increase in transposase-associated ARGs reflected that the dissemination of ARGs in the earthworm gut may be mediated via horizontal gene transfer during the incubation.

In contrast to the control and the nitrate treatment, the nitrite amendment induced a wider range of resistance genes (Figure 3). Because nitrite is an intermediate in the denitrification process, it may contribute to the microbial community’s capacity to harbor an increased number of resistance mechanisms [48,49]. Research by Wonoputri et al. supports the assertion that nitrite can cause cell membrane deterioration [50]. Their findings indicated that treatment of certain microbial cultures with nitrite resulted in membrane disruption, characterized by increased cell surface roughness and degradation due to lipid peroxidation [50]. Earthworms, specifically *Eisenia fetida*, are susceptible to these agents within their gut microbiome, leading to potential ARG mobilization during stress induced by nitrite toxicity [51]. In the microbial community, the nitrite reduction pathway from nitrite to N_2_O was active, and multidrug resistance genes like *acrB* and *mepA* were strongly positively correlated with *Sarcina* and *Anaerosporobacter* [52]. The greater diversity of ARGs observed in this treatment was due to the involvement of these genera in nitrite reduction (Figure 4). This result is consistent with that of earlier research, which indicates that nitrite exposure might promote the spread of resistance genes, perhaps as a result of the microbial community’s enhanced metabolic adaptability to nitrite stress [30].

### 3.3. Most Denitrification Taxa Contribute to ARG Enrichment and Dissemination

As demonstrated in the results, the abundance of denitrification-related bacteria in the earthworm gut was influenced by both nitrate and nitrite treatments. Denitrifiers such as *Aeromonas*, *Raoultella*, *Enterobactor* and *Bacillus* were abundant in the gut microbiome under nitrate and nitrite amendments (Figure 4 and Figure 5). In particular, genera such as *Aeromonas* and *Bacillus* showed strong correlations with resistance to aminoglycosides and glycopeptides (Figure 4 and Figure 5). This indicates that they not only play key roles in nitrogen cycling but also serve as hosts for various ARGs, facilitating their spread within the gut environment. This aligns with findings that the coexistence of ARGs and nitrate reduction genes supports denitrifiers in enhancing antibiotic resistance to environmental stress [44]. This connection is consistent with the mechanism of co-selection, where exposure to nitrite causes shifts in microbial community structure and ARG abundance due to increased horizontal gene transfer (Figure S4). In the nitrite amended gut, such selection would favor denitrifiers that also contain nitrogen cycle genes and ARGs. Because of its genomic fluidity and relatively higher abundance of the mobile genetic element, *Bacillus* could be a critical center in adaptive modifications in ARGs [53]. In addition, *Proteobacteria*, the dominant phylum in the earthworm gut across all treatments, was found to be significantly enriched under the nitrate and nitrite treatments [54]. These genera have been previously associated with both nitrogen reduction and ARG propagation [13,55,56]. The potential of denitrifying genera was highlighted by their strong associations with a number of ARGs, including *vanYd* (glycopeptide) and *aac(6′)-Ib* (aminoglycoside) [34,57]. This finding is consistent with earlier research, which found these genera to be important in the spread of ARGs, particularly in settings with high nitrogen concentrations [53]. Denitrifying bacteria by acquiring ARGs in the earthworm gut confers a defensive advantage and allows them to resist environmental stress and maintain nitrogen cycling. Moreover, nitrite-induced reactive nitrogen species trigger nitrosative stress [51,58]. This stress, coupled with nitrate-induced increases in cell membrane permeability, probably further facilitate the dissemination of ARGs [59,60]. This co-existence of denitrification genes and ARGs enables denitrifiers in the earthworm gut to adapt to and maintain their function under nitrate and nitrite stress, facilitating their survival in nitrogen-enriched environments.

## 4. Materials and Methods

### 4.1. Collection of Earthworms and Microcosms

*Pheritima gullelmi* earthworm adults were collected from Tongling, China. The average local temperature was 25–32 °C, and *P. guillelmi* were collected from soil at a depth of 10–30 cm. Earthworms were collected from this site because of the dense vegetation, farmyards, and frequent fertilizer use, all of which are known to contribute to the spread of antibiotic resistance genes. The process we followed for collecting and analyzing earthworm gut content adhered to the guidelines provided by Schulz (2015) [61]. To ensure clean samples, earthworms were first carefully washed and rinsed with ice-cold deionized water to remove any outside impurities. The specimens were immersed in pure ethanol for one minute in order to sterilize them. Following their exposure to ethanol, the earthworms were rinsed three times with phosphate buffer (0.01 M, pH 7.2) in order to remove any last bits of soil and microorganisms from their bodies.

The earthworms were carefully incised with sterile scissors to obtain the gut contents, which were then placed in 50 mL sterile serum bottles with 10 mL of sterile nitrogen-purged anoxic sodium phosphate buffer (0.1 M). The homogenate of gut contents (from about 40 earthworms) was added to 50 mL serum bottles and stirred well. Three replicates were set up for each comparison to verify the repeatability of the results. The serum bottles were then capped with butyl rubber stoppers, and incubated under dark conditions at 30 °C for 3 days to allow them to become depleted of residual oxygen. This whole process was set up to work under sterile conditions.

Homogenized gut content aliquots (4 mL) were transferred into three 120 mL serum bottles containing 40 mL of evacuated basic medium (N_2_/CO_2_, 80/20%). The basic medium (pH 6.8–7.2) contained MgCl_2_.6H_2_O (0.4 g L^−1^), CaCl_2_.H_2_O (0.1 g L^−1^), NH_4_Cl (0.027 g L^−1^), KH_2_PO_4_ (0.6 g L^−1^), vitamin solution (1 mL L^−1^), trace element solutions (1 mL L^−1^), and bicarbonate buffer (30 mM, to maintain the pH). The vitamin solution, trace elements and bicarbonate buffer were added from stock solutions [62] and were filtered with 0.22 μm filters. All serum bottles were incubated in a precisely controlled 30 °C incubator in the dark. Incubation was further divided into three setups. Glucose was added to the basic medium as an exogenous source of organic carbon.

Four treatments were established: (1) the control, in which only the guts were added to basic media (Ck), (2) the nitrate amendment, in three concentrations (T1 = 0.5 mM, T2 = 2 mM, T3 = 5 mM), and (3) the nitrite amendment, in three concentrations (T4 = 0.1 mM, T5 = 0.5 mM, T6 = 1 mM). Microcosms were set up in triplicate and incubated at 28 °C in the dark for four days. Sampling was undertaken on days 1, 2, 3, and 4.

### 4.2. Analytical Methods

The concentrations of nitrate, nitrite, and organic acid in the samples were analyzed using ion chromatography Dionex ICS-3000 ion chromatography system (Thermo Fisher Scientific), Sunnyvale, California, USA [63]. The serum vials were shaken for a minute, and then filtered through a 0.22 um filter. The filtered samples were diluted with sterile deionized water in a 1:4 (*w*/*v*) ratio. Glucose quantification was performed using the anthrone method [64], with meticulous modifications to the original protocol. In the improved method, glucose was first dried at 80 °C for 6 h to achieve a constant weight. Following this, a standard solution was prepared. For the anthrone reagent, 50 mg of anthrone was weighed and dissolved in 70% concentrated sulfuric acid by volume. This meticulous procedure ensured enhanced accuracy in determining glucose concentration. Nitrous oxide (N_2_O) was detected using gas chromatography [65]. Before determination, all serum vials were shaken vigorously for 1 min to ensure gas homogeneity before analysis.

### 4.3. Bacterial 16S rRNA Gene Amplification, Illumina Sequencing and Data Processing

Genomic DNA was extracted from samples using the TIANamp DNA kit (TIANamp DNA kit (TianGen Biotech, Beijing, China)) according to the manufacturer’s protocol. The quality of the DNA was assessed with a NanoDrop ND-1000 (Thermo Scientific, Wilmington, DE, USA). For PCR amplification, a reaction mix (25 μL) was meticulously prepared. The V4 and V5 regions of bacterial 16S rRNA genes were amplified. The forward primer was 515F *(5′-GTGCCAGCMGCCGCGG-3′)*, and the reverse primer consisted of a six bp barcode and 907R *(5′-CCGTCAATTCMTTTRAGTTT-3′)*. The amplification process started with initial denaturation at 95 °C for 2 min, followed by denaturation (30 cycles) for 30 s at 95 °C, annealing at 55 °C for 30 s, and extension at 72 °C for 30 s. The final step of extension occurred at 72 °C for 10 min. The amplicons were purified, quantified, pooled, and subsequently sequenced on an Illumina Novseq 6000 platform at Shanghai Biozeron Technology Co., Ltd. (Shanghai, China).

### 4.4. qPCR Amplification

The denitrification genes (*narG*, *nirK*, *nirS* and *nosZ*) were quantified. The qPCR protocol was performed in a Fast Optical 96-well plate format. Each biological sample was run in triplicate to assess the quality of the results. The primer sequences of the target genes were chosen from a published study [66]. In particular, *narG* was amplified by using the *narG1960f/QICNarG-2650r* primer pair (*5-TAY GTS GGS CAR GAR AA-3* for the forward primer and *5-TTY TCR TAC CAB GTA GC-3* for the reverse primer) and *nosZ* was amplified using *nosZF/nosZR* primers *(5-CCCGCTGCACACCRCCTTCGA-3* and *5-CGTCGCCSGAGATGTCGATCA-3)* [67,68,69,70] (Table S1).

The reaction mixture for qPCR amounted to 20 μL with 10 μL of 2× SuperReal PreMix Plus, 0.7 μL of primer (10 μM of each primer), 0.7 μL of 50× ROX Reference Dye, 2 μL of templates DNA and 6.2 μL of ddH_2_O. Preliminary denaturation at 95 °C was performed for 8 min, after which 35 cycles of amplification were performed. The cycles consisted of denaturation at 95 °C for 1 min, annealing at a temperature specific to the gene for 30 s, and elongation at 72 °C for 2 min. The extension of the terminals was performed at 72 °C and took 10 min. The annealing temperatures differed by gene: the temperature for *narG* was 55 °C, that for *nosZ* was 58 °C, that for *nirK* was 57 °C, and that for *nirS* was 54 °C. A final extension was performed at 72 °C after the 5 min amplification cycles. These standard curves were used to automatically determine the abundance of *nirK*, *nirS*, *narG*, and *nosZ*, and the *16S rDNA* gene served as a reference gene.

### 4.5. ARGs Quantification

The abundance of ARGs and transposase in the gut was estimated using qPCR, with a reactor from Qubit^®^. A total of 48 primer pairs, each meticulously designed to target specific ARGs, were used. All amplifications were performed in triplicate. The detection limit is 31 for the threshold cycle (Ct). The relative abundances of functional genes were calculated after normalization by *16S rRNA* gene copy numbers, i.e., the functional gene copy number divided by the *16S rRNA* gene copy number. A total of 23 ARGs were quantified and mentioned in this work (Table S2). Denitrification taxa were identified using the NCBI database to analyze the host taxa of the ARGs.

### 4.6. Statistical Analysis

Analysis of variance (ANOVA) was carried out with SPSS version 18.0 (SPSS Inc., Chicago, IL, USA). Statistical significance was evaluated by ANOVA (*p* < 0.05). Plots were generated using OriginPro 2024 (OriginLab Corporation, Northampton, MA, USA), R software, with the ggplot2 package. Bubble figure and correlation analysis was performed using Mantel analysis, with significance denoted at *p* < 0.05 in R 4.3.1, via the linkET package. The igraph package was used for network analysis. Statistical significance of the diversity and relative abundances of bacterial communities and ARGs between samples were determined using a one-way analysis of variance and Tukey’s HSD test.

## 5. Conclusions

In conclusion, both nitrate and nitrite amendments significantly influenced the dissemination of antibiotic resistance genes (ARGs) in the earthworm gut. Among the treatments, nitrite amendments, particularly at 1 mM, demonstrated a greater role in promoting ARG dissemination compared to nitrate amendments. There was a marked increase in the relative abundance of sulfonamide, beta-lactam, and aminoglycoside resistance genes in the nitrite treatment during the incubation. In addition, the 5 mM nitrate treatment exhibited the highest abundances of denitrifiers, including *Aeromonas*, *Bacillus*, and *Enterobacter*, which acted as co-hosts for ARGs, particularly under elevated nitrate conditions. Denitrifying taxa including *Aeromonas*, *Anaerosporobacter*, *Raoultella*, and *Sarcina* have been identified as important players in the spread of ARGs. Our results indicated that the coexistence of denitrifiers and ARGs triggers the bacterial defense mechanism. Nitrate and nitrite amendments activate the stress response with the upregulation of antibiotic resistance genes and the activation of transposases. The presence of denitrifiers with antibiotic resistance genes further supports the idea that they are significant hosts of antibiotic resistance genes. The findings underscore the role of denitrifying bacteria as key mediators of ARG dissemination, with nitrate and nitrite acting as critical factors in shaping both nitrogen cycling and ARG spread in soils.

## Data Availability

Data have been uploaded to the NCBI, with the accession no. PRJNA1370075.

## Supplementary Materials

The following supporting information can be downloaded at https://www.mdpi.com/article/10.3390/ijms27020797/s1.
