# Supplementary material for: Denitrifiers Make Great Contribution to Antibiotic Resistance Genes Dissemination in the Gut of Earthworms
Source: Int J Mol Sci. 2026 Jan 13;27(2):797. doi: 10.3390/ijms27020797 (PMC12840990; doi:10.3390/ijms27020797)
Supplement: Supplementary file 1 [file ijms-27-00797-s001.zip › ijms-4035062-supplementary/Supplementary data/Supplementary data file.pdf]

Abbreviations

The following abbreviations are used in this manuscript

ARGs	Antibiotic resistance genes
qPCR	quantitatice ploymerase chain reaction
16s rRNA	16s ribosomal RNA
NO_3^-	Nitrate
NO_2^-	Nitrite
NO	Nitric oxide
N_2O	Nitrous oxide

Table S1 Nitrate reduction primers and qPCR conditions

Targeted gene	Primer ID	Primers	Anealing temp.
narG	*narG-1960F*	TAYGTSGGCARGARAA	55°C
	narG-2650R	TTYTCRTACCABGTAGC	
nirK	*nirKF*	GGMATGGTKCCSTGGCA	57°C
	nirKR	AACTTGCCGTVGYCAGAC	
nirS	*nirS-832F*	TAYCACCCSGARCCGCGCGT	54°C
	NirS-1606R	AGKCGTTGACTTKCCGGTCG	
nosZ	*nosZF*	CCCGCTGCACACCRCCTTCGA	58°C
	nosZR	CGTGCCSGAGATGTCGATCA	

Table S2 ARGs primers

Targeted gene	Primer F	Primer R	Catogory
aac(6′)-Ib	CGTCGCCGAGCAACTTG	CGGTACCTTGCCTCTCAAACC	Aminoglycoside
spcN	GCTATGTGCTGGTGGACTGG	GGAACCACTCGACGAACTCG	Aminoglycoside
strB	GCTCGGTCGTGAGAACAATCT	CAATTTCGGTCGCCTGGTAGT	Aminoglycoside
aadA2-1	ACGGCTCCGCAGTGGAT	GGCCACAGTAACCAACAAATCA	Aminoglycoside
ampC/blaDHA	TGGCCGCAGCAGAAAGA	CCGTTTTATGCACCCAGGAA	Beta Lactam
blaMOX/blaCMY	CTATGTCAATGTGCCGAAGCA	GGCTTGTCCTCTTTCGAATAGC	Beta Lactam
vanYD	AAGGCGATACCCTGACTGTCA	ATTGCCGGACGGAAGCA	Glycopeptide
mphA	TCAGCGGGATGATCGACTG	GAGGGCGTAGAGGGCGTA	MLSB
cmlA1	TAGGAAGCATCGGAACGTTGAT	CAGACCGAGCACGACTGTTG	Multidrug
floR	AACCCGCCCTCTGGATCA	GCCGTCGAGAAGAAGACGAA	Multidrug
mepA	ATCGGTCGCTCTTCGTTCAC	ATAAATAGGATCGAGCTGCTGGAT	Multidrug
msr(A)	CTGCTAACACAAGTACGATTCCAAAT	TCAAGTAAAGTTGTCTTACCTACACCATT	Multidrug
tolC	GGCCGAGAACCTGATGCA	AGACTTACGCAATTCCGGGTTA	Multidrug
ttgB	TCGCCCTGGATGTACACCTT	ACCATTGCCGACATCAACAAC	Multidrug
acrB	AGTCGGTGTTCGCCGTTAAC	CAAGGAAACGAACGCAATACC	Multidrug
acrA	GGTCTATCACCCTACGCGCTATC	GCGCGCACGAACATACC	Multidrug
sul1	GCCGATGAGATCAGACGTATTG	CGCATAGCGCTGGGTTTC	Sulfonamide
sul2	TCATCTGCCAAACTCGTCGTTA	GTCAAAGAACGCCGCAATGT	Sulfonamide
tetB	AGTGCGCTTTGGATGCTGTA	AGCCCCAGTAGCTCCTGTGA	Tetracycline
tetD	AATTGCACTGCCTGCATTGC	GACAGATTGCCAGCAGCAGA	Tetracycline
tetL	ATGGTTGTAGTTGCGCGCTATAT	ATCGCTGGACCGACTCCTT	Tetracycline
tetQ	CGCCTCAGAAGTAAGTTCATACACTAAG	TCGTTCATGCGGATATTATCAGAAT	Tetracycline
tnpA-2	CCGATCACGGAAAGCTCAAG	GGCTCGCATGACTTCGAATC	Transposase

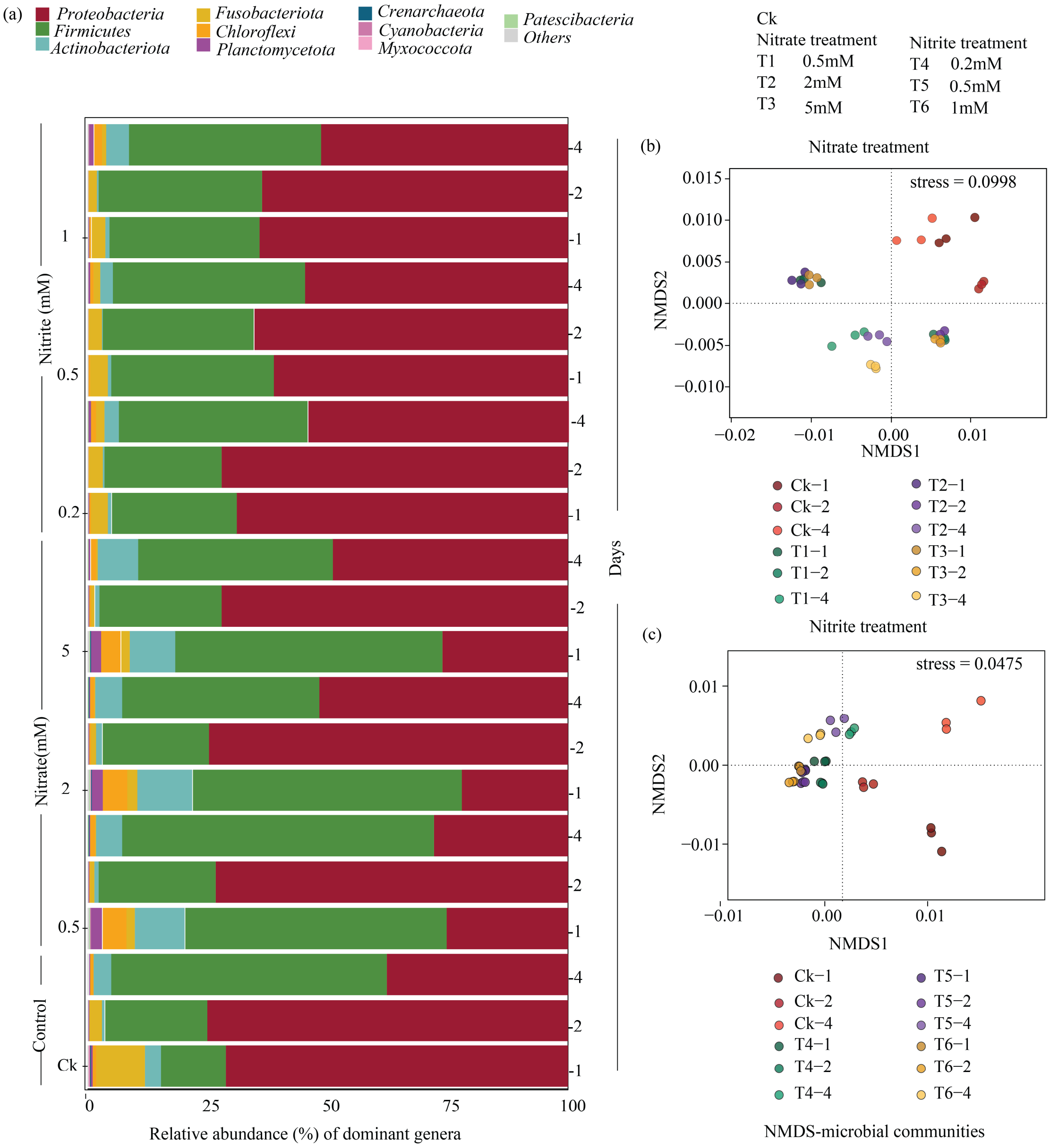

Figure S1 Effects of nitrate and nitrite amendments in microbial diversity and composition, (**a**) indicate the relative abundance (%) of dominant phyla while (**b** and **c**) represents the NMDS. Stress values are mentioned in the figure. T1, T2, T3 are the nitrate treatments (0.5, 2 and 5 mM), T4, T5, T6 are the nitrite treatments (0.2, 0.5 and 1mM)

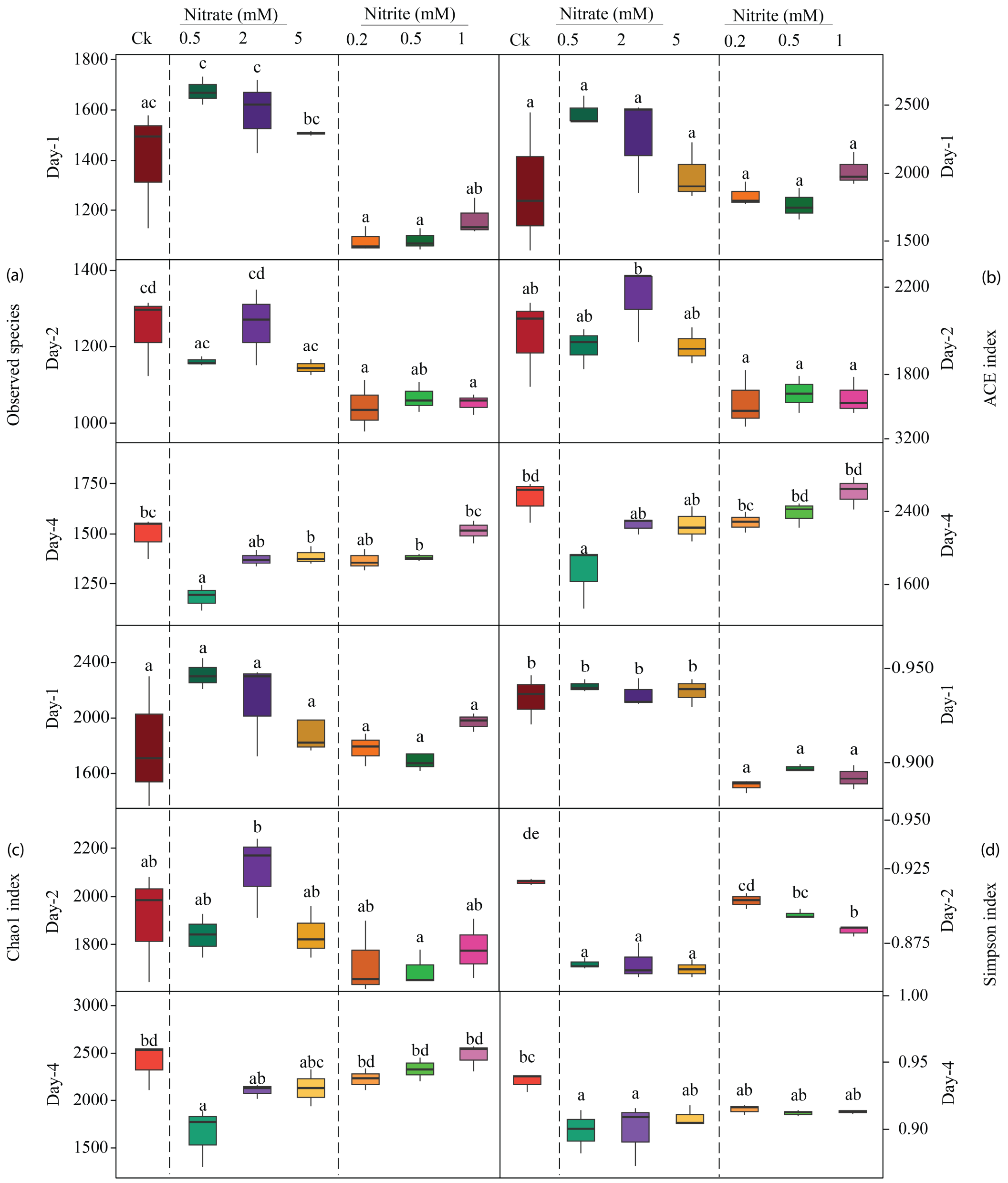

Figure S2. *Effects of nitrate and nitrite amendments on microbial diversity indices.* (**a**) Boxplots indicate the observed species count, (**b**) ACE index, (**c**) Chao1 index, and (**d**) Simpson index across different treatments at days 1, 2, and 4. Letters above the boxes denote significant differences. T1, T2, T3 represent the nitrate treatments (0.5, 2, and 5 mM), and T4, T5, T6 represent the nitrite treatments (0.2, 0.5, and 1 mM)

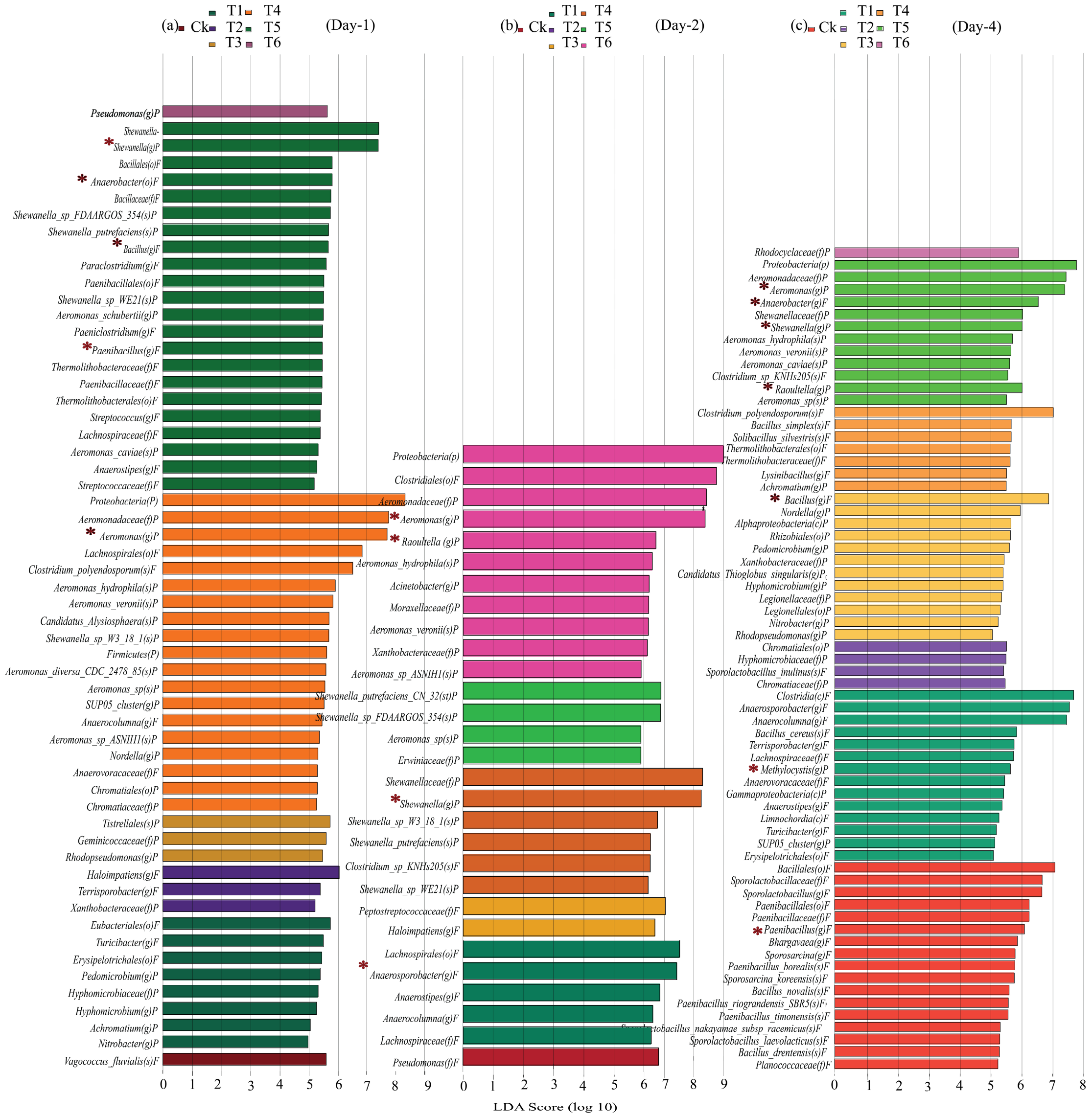

Figure S3. *Differentially abundant microbial taxa in response to nitrate and nitrite amendments identified by LEfSe analysis.* (**a**) day 1, (**b**) day 2, and (**c**) day 4 LDA scores of microbial taxa across different nitrate and nitrite treatments. Taxa significantly enriched in each treatment group are indicated with an asterisk. T1, T2, T3 represent the nitrate treatments (0.5, 2, and 5 mM), and T4, T5, T6 represent the nitrite treatments (0.2, 0.5, and 1 mM)

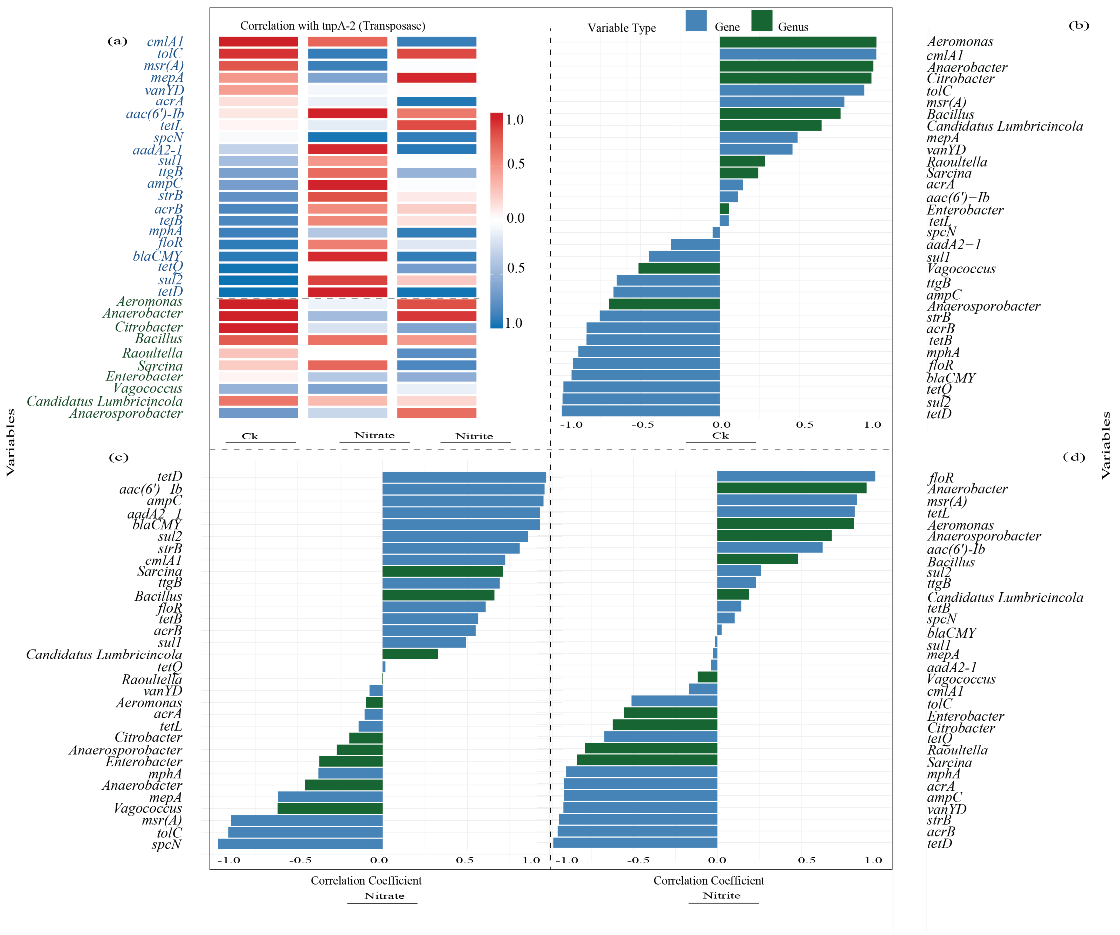

Figure S4. Pearson Correlation analysis of the transposase gene *tnpA-2* with ARGs and genus under different nitrogen treatments. (a) Heatmap showing Pearson correlation coefficients between *tnpA-2* and selected antibiotic resistance genes and microbial genera under control (CK), nitrate, and nitrite treatments. Bar plots illustrating the correlation strength between *tnpA-2* and individual antibiotic resistance genes (blue bars) and microbial genera (green bars) under (b) control (CK), (c) nitrate, and (d) nitrite treatments. Positive and negative values indicate positive and negative correlations, respectively, highlighting treatment-specific associations between *tnpA-2*, resistance genes, and microbial taxa
