# Supplementary figures and images for: Denitrifiers Make Great Contribution to Antibiotic Resistance Genes Dissemination in the Gut of Earthworms
Source: Int J Mol Sci. 2026 Jan 13;27(2):797. doi: 10.3390/ijms27020797 (PMC12840990; doi:10.3390/ijms27020797)

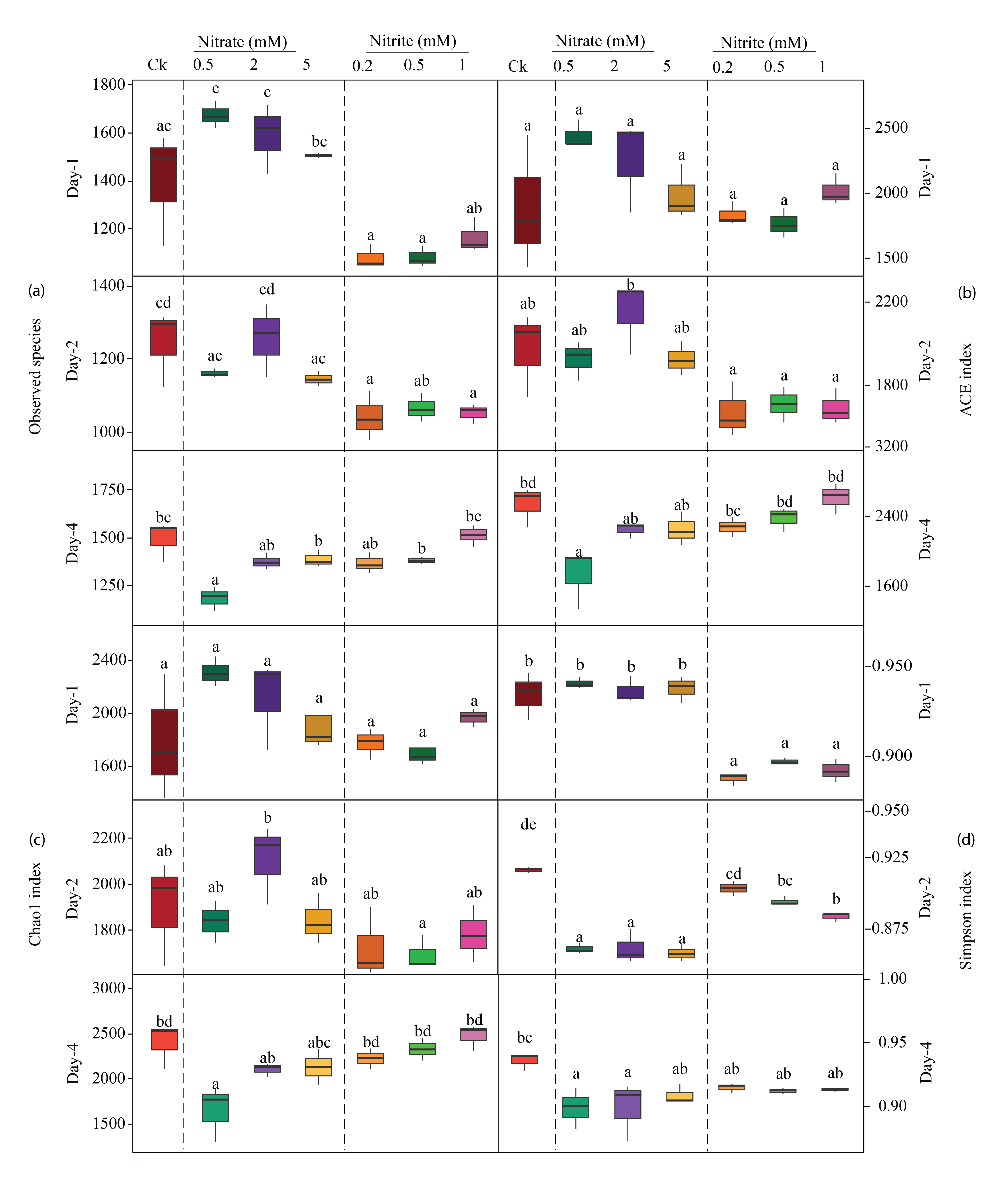

Supplement: Supplementary file 1 [file ijms-27-00797-s001.zip › ijms-4035062-supplementary/Supplementary data/supplementary figure S2 (index merged).png]

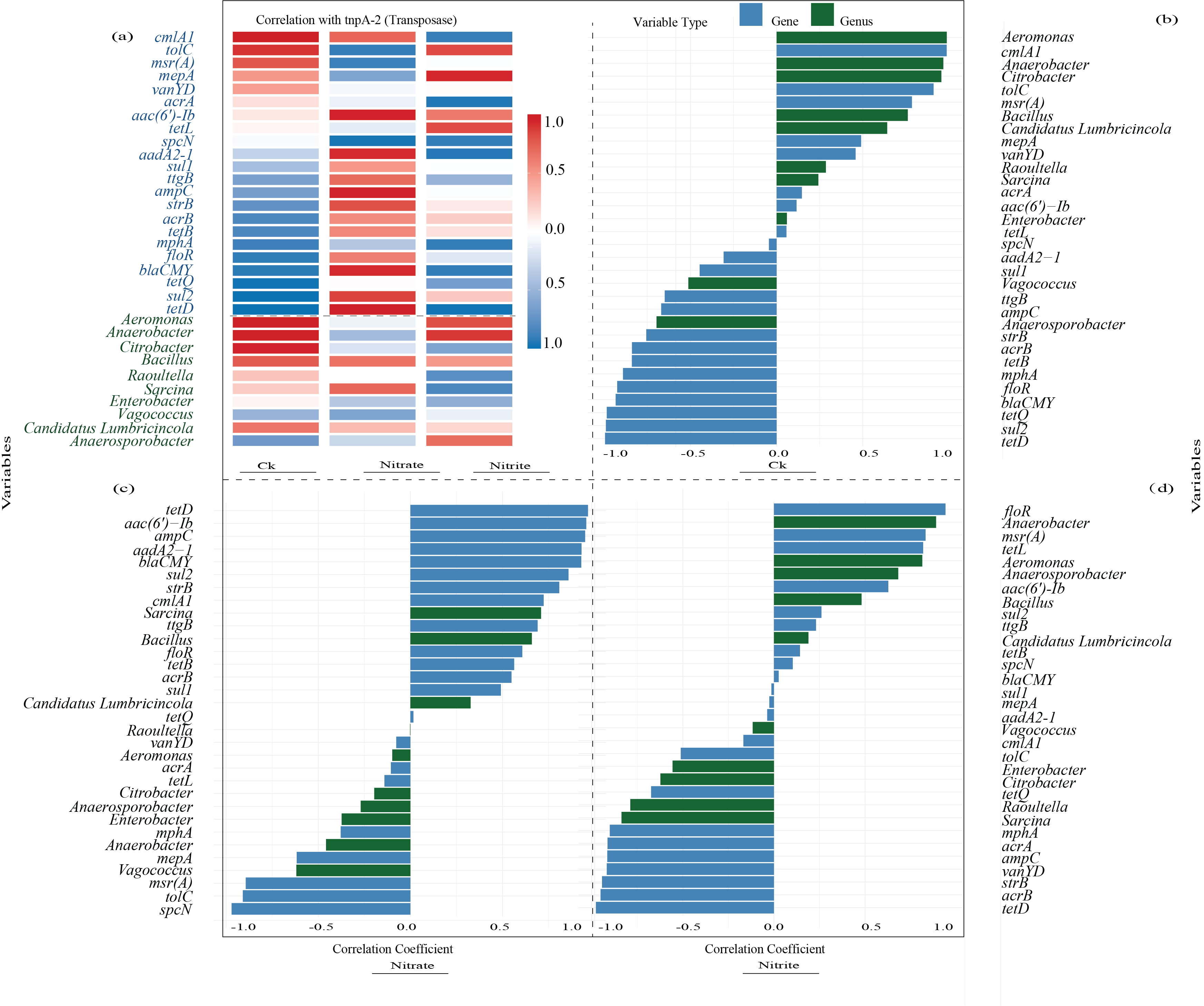

Supplement: Supplementary file 1 [file ijms-27-00797-s001.zip › ijms-4035062-supplementary/Supplementary data/Supplementary figure S4.png]

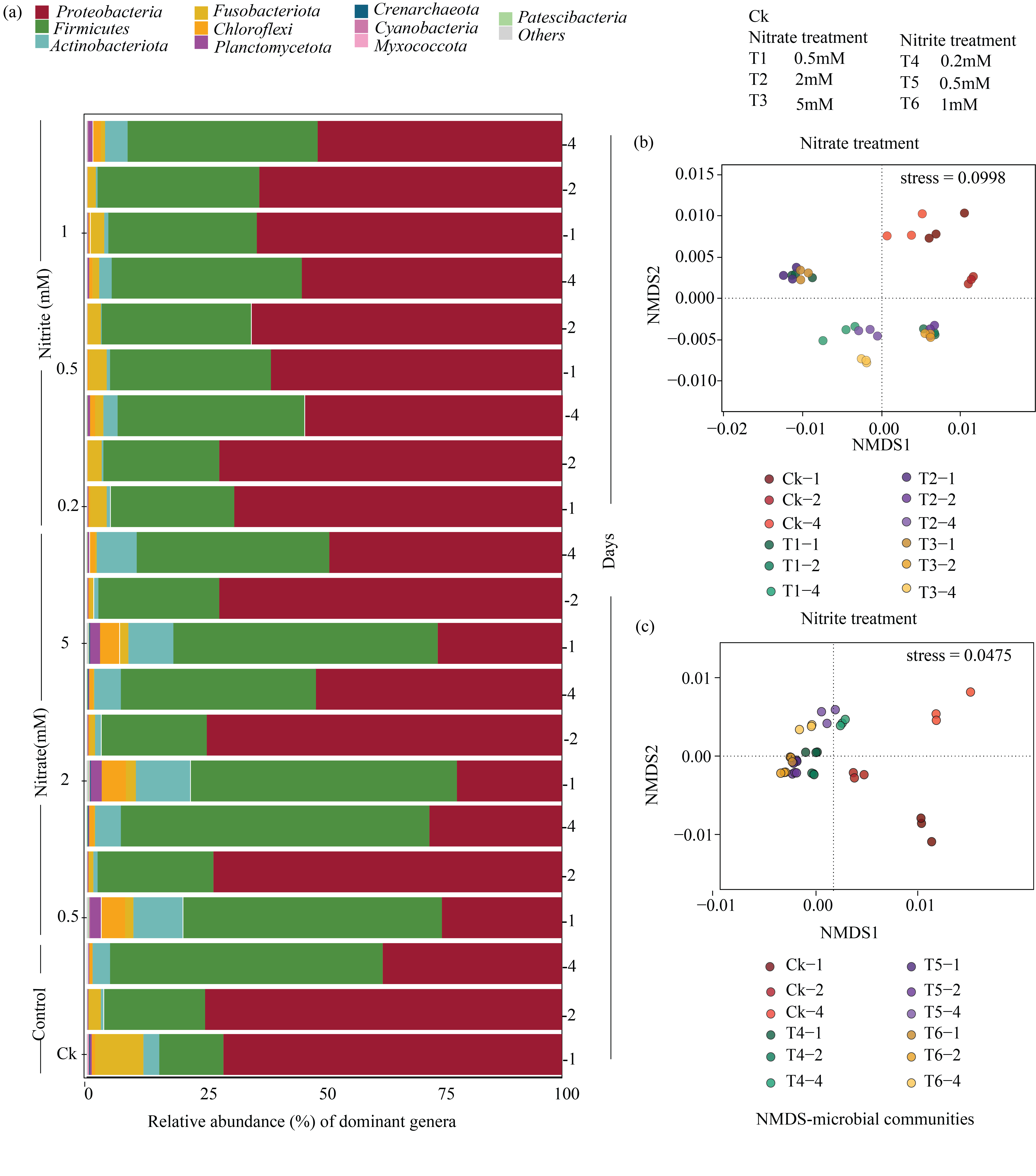

Supplement: Supplementary file 1 [file ijms-27-00797-s001.zip › ijms-4035062-supplementary/Supplementary data/Supplemntary figure S1 (phylum and NMDS).png]
